# Apatinib inhibits the growth of small cell lung cancer via a mechanism mediated by VEGF, PI3K/Akt and Ki‐67/CD31

**DOI:** 10.1111/jcmm.16926

**Published:** 2021-09-30

**Authors:** Ning Zhong, Wei Zhuang, Qian Huang, Qiang Wang, Wenjian Jin

**Affiliations:** ^1^ Department of Geriatric Oncology Jiangxi Provincial Tumor Hospital Nanchang China; ^2^ Jiangxi Health Vocational College Nanchang China; ^3^ Department of Abdominal Surgery Jiangxi Provincial Tumor Hospital Nanchang China; ^4^ Department of Oncology the People’s Hospital of Ruijin City Ruijin China

**Keywords:** apatinib, small cell lung cancer, targeted therapy, tumour angiogenesis, vascular endothelial growth factor, vascular endothelial growth factor receptor 2

## Abstract

This study aimed to investigate the anti‐tumour effect of apatinib on extensive‐stage small cell lung cancer (SCLC) and elucidate the associated mechanisms. NCI‐H345 cells were selected as model cells because of high expression of vascular endothelial growth factor (VEGF), VEGF receptor 2 (VEGFR2) and phosphorylated‐VEGFR2 (pVEGFR2). Cells were exposed to recombinant human VEGF (rhVEGF) and apatinib. Cells were then divided into eight groups, namely, control, rhVEGF, apatinib, rhVEGF+apatinib, serum‐free medium (SM), SM+rhVEGF, SM+apatinib and SM+rhVEGF+apatinib. In comparison with the control group, cell proliferation *in vitro* in apatinib, SM, SM+apatinib and SM+rhVEGF+apatinib groups was inhibited, particularly in SM+apatinib group. The effect of apatinib on tumour growth *in vivo* was investigated using a mouse xenograft tumour model. In comparison with the control group, tumour sizes were reduced in apatinib‐treated group on days 34 and 37. Immunohistochemical and immunofluorescence staining revealed that VEGF, pVEGFR2, PI3K, AKT, p‐ERK1/2, Ki‐67 and CD31 in the tumour cells of apatinib‐treated group were downregulated compared with control group. Haematoxylin and eosin staining revealed that apatinib promoted the necrosis of SCLC cells in vivo. In conclusion, apatinib inhibited the growth of SCLC cells by downregulating the expression of VEGF, pVEGFR2, p‐PI3K, p‐AKT, p‐ERK1/2, Ki‐67 and CD31.

## 1 INTRODUCTION

Small cell lung cancer (SCLC) is a type of poorly differentiated neuroendocrine tumour characterized by rapid growth, early metastasis, and sensitivity to radiotherapy and chemotherapy.^1^ SCLC accounts for 14% of lung cancer cases and is closely associated with smoking. In fact, >95% of patients with SCLC have a history of smoking, causing high‐frequency gene mutations.^1^ Several drugs have been developed for the treatment SCLC, but satisfying outcomes have not been achieved.^1^ Moreover, while the short‐term objective remission rate of SCLC is high, relapse and drug resistance occur in the majority of patients within 6 months of remission, resulting in poor long‐term efficacy. Furthermore, the short doubling time of SCLC cells and early distant metastasis complicate the accurate diagnosis and adequate treatment. Patients with extensive stage‐SCLC (ES‐SCLC) are particularly susceptible to recurrence after 4–6 cycles of platinum‐based first‐line chemotherapy, and the efficiency of current second‐line agents is poor.^2^, ^3^, ^4^ Recent studies have reported novel therapeutic agents such as pegfilgrastim and irinotecan for ES‐SCLC.^5^, ^6^, ^7^

SCLC tumours are characterized with high microvessel density and a rich blood supply.^8^ Vascular endothelial growth factor (VEGF) is upregulated in the serum of the majority of patients with SCLC and is negatively correlated with the sensitivity of chemotherapy and survival time.^8^ In the past decades, VEGF and the VEGF receptor (VEGFR) have served important roles in tumorigenesis.^9^, ^10^, ^11^ The suppression of VEGF expression by VEGF neutralizing antibody inhibits tumour growth and metastasis,^8^ and an increasing number of drugs, such as bevacizumab and sunitinib, which target VEGF and VEGFR, have been developed.^9^, ^10^, ^11^, ^12^

Apatinib is a highly selective VEGFR2 inhibitor, which inhibits the angiogenesis of nasopharyngeal carcinoma, non‐Hodgkin lymphoma and small‐cell carcinoma of oesophagus.^13^, ^14^, ^15^, ^16^ The clinical efficacy of apatinib in advanced tumours, including relapsed or refractory non‐Hodgkin lymphoma, metastatic gastric cancer, colorectal cancer, non‐SCLC (NSCLC), neuroendocrine tumours and mesothelioma, has been demonstrated in several clinical trials.^14^, ^15^ Therefore, apatinib may be a suitable therapeutic agent for the maintenance treatment of patients with extensive‐stage (ES)‐SCLC. However, to the best of the authors' knowledge, the efficacy of apatinib as maintenance therapy in ES‐SCLC has not been reported, and the efficacy, safety and mechanisms of apatinib monotherapy in ES‐SCLC deserve further investigation. Accordingly, the present study aimed to investigate the anti‐tumour effect of apatinib in ES‐SCLC by using in vivo and in vitro models.

## 2 MATERIALS AND METHODS

### 2.1 Cell lines, animals and apatinib

The human SCLC cell lines (NCI‐H345 and NCI‐H446) were obtained from BeNa Culture Collection (BNCC). Cells were cultured in complete RPMI‐1640 medium (KeyGen Biotech) containing 10% foetal bovine serum (Thermo Fisher Scientific) at 37°C in an incubator containing 5% CO_2_.

Eight male nude mice (age, four weeks; weight: 16–20 g) were obtained from Hunan SJA Laboratory Animal Co., Ltd. Animals were housed at 22–25°C and had free access to food and water. No signs of necrosis, infection or ulcer were observed during the experiment. Experiments could not affect the normal activities and eating behaviour of the mice. The study protocol was approved by the Ethics Committee of Jiangxi Provincial Tumor Hospital.

Apatinib mesylate tablets (250 mg/tablet, calculated as apatinib) were obtained from Jiangsu Hengrui Medicine Co., Ltd. For in vitro use, tablets were dissolved in dimethyl sulphoxide to obtain a 50 mM stock solution, which was subsequently diluted in RPMI‐1640 medium to produce the required working solutions. For *in vivo* use, the tablet was dissolved in normal saline.

### 2.2 Cell screening and rhVEGF treatment

The levels of VEGF, VEGFR2, and pVEGFR2 in NCI‐H345 and NCI‐H446 cells were examined by Western blot analysis to select the ideal cell model. NCI‐H345 and NCI‐H446 cells were subsequently treated with 30 ng/ml recombinant human VEGF (rhVEGF; cat. no 10542‐H08H; Sino Biological) for 0, 30, 60 and 120 min at 37°C. The levels of VEGFR2 and pVEGFR2 in the cells were examined by Western blot analysis to identify the optimal exposure time.

### 2.3 Western blot analysis

Cells were lysed in radioimmunoprecipitation assay buffer (Beyotime) at 4°C for 30 min and centrifuged for 10 min at 4°C and 9000 ×*g*. Total protein concentration was quantified using bicinchoninic acid assay. Proteins were subsequently denatured by boiling for 5 min, and 30 ng protein/lane was separated via SDS‐PAGE (10%) for 1.5 h. The separated proteins were transferred to polyvinylidene fluoride membranes (EMD Millipore) and blocked for 1 h at room temperature with 2% bovine serum albumin (BSA, Beyotime). The membranes were incubated with primary antibodies against VEGF (1:1,000, cat. no. ab32151; Abcam), VEGFR2 (1:1,000, cat. no. ab11939; Abcam), phospho‐VEGFR2 (1:500, bs‐2674R; Bioss) and GAPDH (1:1,000, cat. no. TA‐08; ZSGB‐Bio) overnight at 4°C. Following the primary incubation, membranes were incubated with peroxidase‐conjugated secondary antibodies (1:2,500, cat. nos. ZB‐2305 and ZB‐2301; ZSGB‐Bio) for 1–2 h at room temperature. Protein bands were visualized using the SuperSignal^®^ West Pico chemiluminescent substrate (Thermo Fisher Scientific, Inc.) and a ChemiDoc^TM^ XRS+gel imaging system (Bio‐Rad Laboratories, Inc.). Protein expression was quantified using Quantity One software (version 4.62; Bio‐Rad Laboratories, Inc.) with GAPDH as the loading control.

### 2.4 Cell proliferation in vitro

NCI‐H345 cells were seeded at a density of 10,000 cells per well in a 96‐well plate and cultured at 37°C and 5% CO_2_ for 24 h. The cells were treated with increasing concentrations of apatinib (50, 100, 150, 200 and 250 nM) for different durations (24, 48 and 72 h). The concentration of apatinib was selected as previously described (17). A total of 10 μL of cell counting kit‐8 (CCK‐8) solution were added per well, and the cells were incubated for 2 h. Cell viability (%) was calculated by measuring the absorbance at a wavelength of 570 nm by using a microplate reader (cat. no. RT‐6100; Rayto).

In addition, the cells were divided into eight groups as follows: (i) control (without any treatment); (ii) rhVEGF (cells were treated with 30 ng/ml rhVEGF for 60 min); (iii) apatinib (cells were treated with 250 nM apatinib for 60 min); (iv) rhVEGF+apatinib (cells were treated with 30 ng/ml rhVEGF and 250 nM apatinib for 60 min); (v) serum‐free medium (cells were cultured in serum‐free medium for 12 h); (vi) serum‐free medium+rhVEGF (cells were cultured in serum‐free medium for 12 h and then treated with 30 ng/ml rhVEGF for 60 min); (vii) serum‐free medium+apatinib (cells were cultured in serum‐free medium for 12 h and then treated with 250 nM apatinib for 60 min); and (viii) serum‐free medium+rhVEGF+apatinib (cells were cultured in serum‐free medium for 12 h and then treated with 30 ng/ml rhVEGF and 250 nM apatinib for 60 min). Cell proliferation was investigated in each group via CCK‐8 assay.

### 2.5 Tumour growth in vivo

Eight nude mice were anaesthetized by inhaling ether. A total of 1 × 10^7^ NCI‐H345 cells in 0.2 ml of normal saline were injected subcutaneously into the right axillary region on day 0. Cerebral state index, bunt suppression, electromyographic and signal quality index were monitored to ensure that the animals were anaesthetized rather than euthanized after exposure to ether. Tumour sizes were calculated approximately twice a week by using the following equation: *a* × *b* × *b*/2, where *a* and *b* represent the long and short diameters of the tumour, respectively. The long and short diameters of the tumour were measured using a Vernier caliper. Mice were randomly divided into the two following groups (*n* = 4 per group): (i) apatinib‐treated group, in which mice received 50 mg/kg apatinib by gavage every day; and (ii) control groups, in which the mice received isovolumetric normal saline by gavage every day. The last tumour measurement was obtained 37 days after the cell injection, and the mice were anaesthetized via intraperitoneal injection of 5% chloral hydrate (400 mg per kg of animal body weight) and euthanized via cervical dislocation. The tumour burden should be less than 10% of the weight of the animal. Tumours were harvested for subsequent investigation.

### 2.6 Immunohistochemical analysis

Tumour tissues were fixed in 4% paraformaldehyde at room temperature for 10 min, embedded in paraffin and cut into sections (4 μm thickness). The sections were dehydrated in 70%, 80% and 90% ethanol solutions and dewaxed in xylene twice and rehydrated. Antigen retrieval was performed by incubating the sections in citrate buffer and heating in a high‐pressure cooker for 2 min. The sections were allowed to cool, washed with phosphate buffer solution (PBS), and incubated in fresh 3% hydrogen peroxide for 10 min at room temperature. The sections were washed thrice with PBS and incubated with 5% BSA at 37°C for 30 min. The sections were incubated with primary antibodies against VEGF (1:1,000, cat. no. ab32151; Abcam), pVEGFR2 (1:1,000, bs‐2674R; Bioss), PI3K (1:1,000, AF5315; ABclonal Technology), p‐PI3K (1:1,000, AP0854; ABclonal Technology), AKT (1:1,000, ab8805; Abcam), p‐AKT (1:1,000, bs‐2720R; Bioss), ERK1/2 (1:1,000, 4370; Cell Signaling), p‐ERK1/2 (1:1,000, bs‐3016R; Bioss) and Ki‐67 (1:1,000, KG22487‐4; KeyGen Biotech) overnight at 4°C. The sections were washed thrice with PBS and incubated with peroxidase‐conjugated secondary antibodies (1:2,500, cat. nos. ZB‐2305 and ZB‐2301; ZSGB‐Bio) for 30 min at 37°C. The sections were subsequently washed with PBS, stained with diaminobenzidine for 5–10 min, rinsed with PBS for 1 min and counterstained in haematoxylin for 3 min. The sections were differentiated in hydrochloric alcohol for 15 s, incubated with bluing buffer (Beyotime) at room temperature for 10 s and rinsed with water for 1 min. Sections were mounted and evaluated using a light microscope (CX41, Olympus Corporation). Semi‐quantitative analysis was performed using ImageJ software (version 1.8.0; National Institutes of Health).

### 2.7 Immunofluorescence analysis

The paraffin sections were dehydrated, dewaxed and rehydrated. Antigen retrieval was performed by incubating the sections in citrate buffer and heating in a high‐pressure cooker for 2 min. The sections were allowed to cool, washed with PBS and incubated with 0.5% Triton X‐100 for 20 min at room temperature. The sections were washed thrice with PBS and incubated in 5% BSA at 37°C for 30 min. The sections were subsequently incubated with primary antibodies against CD31 (1:200, cat. no. ab32457; Abcam) overnight at 4°C. The sections were washed thrice with PBS and incubated with alexa fluor 647‐conjugated goat anti‐rabbit IgG(H+L) (1:500, cat. no. A0468; Beyotime) for 30 min at 37°C in the dark. The sections were stained with 4′,6‐diamidino‐2‐phenylindole at room temperature for 5 min in the dark, rinsed with PBS, mounted with neutral resin and observed under fluorescence microscope (CKX53; Olympus Corporation). Semi‐quantitative analysis was performed using ImageJ software (version 1.8.0; National Institute of Health).

### 2.8 Haematoxylin and eosin staining

Tumour samples were washed, dehydrated in 70%, 80% and 90% ethanol solutions, immersed in equal volumes of absolute alcohol and xylene for 15 min, transparentized in xylene twice and immersed in equal volumes of paraffin and xylene for 15 min. The samples were subsequently embedded in paraffin and cut into 4 μm‐thick sections, which were baked, dewaxed and hydrated. Hydrated slices were immersed in haematoxylin solution for 3 min, differentiated in hydrochloric alcohol for 15 s, and washed and incubated in bluing buffer for 15 s. The sections were subsequently washed with running water and immersed in eosin solution for 3 min, and washed, mounted and examined under a light microscope (CKX41; Olympus Corporation).

### 2.9 Statistical analysis

Data were presented as mean ± standard deviation. Statistical analysis was performed using SPSS software (version 19.0; IBM Corp.). One‐way analysis of variance and Tukey post hoc test were used to compare the different groups. Significant difference was considered at *p* < 0.05.

## 3 RESULTS

### 3.1 Cell line screening and rhVEGF exposure time

**FIGURE 1:**
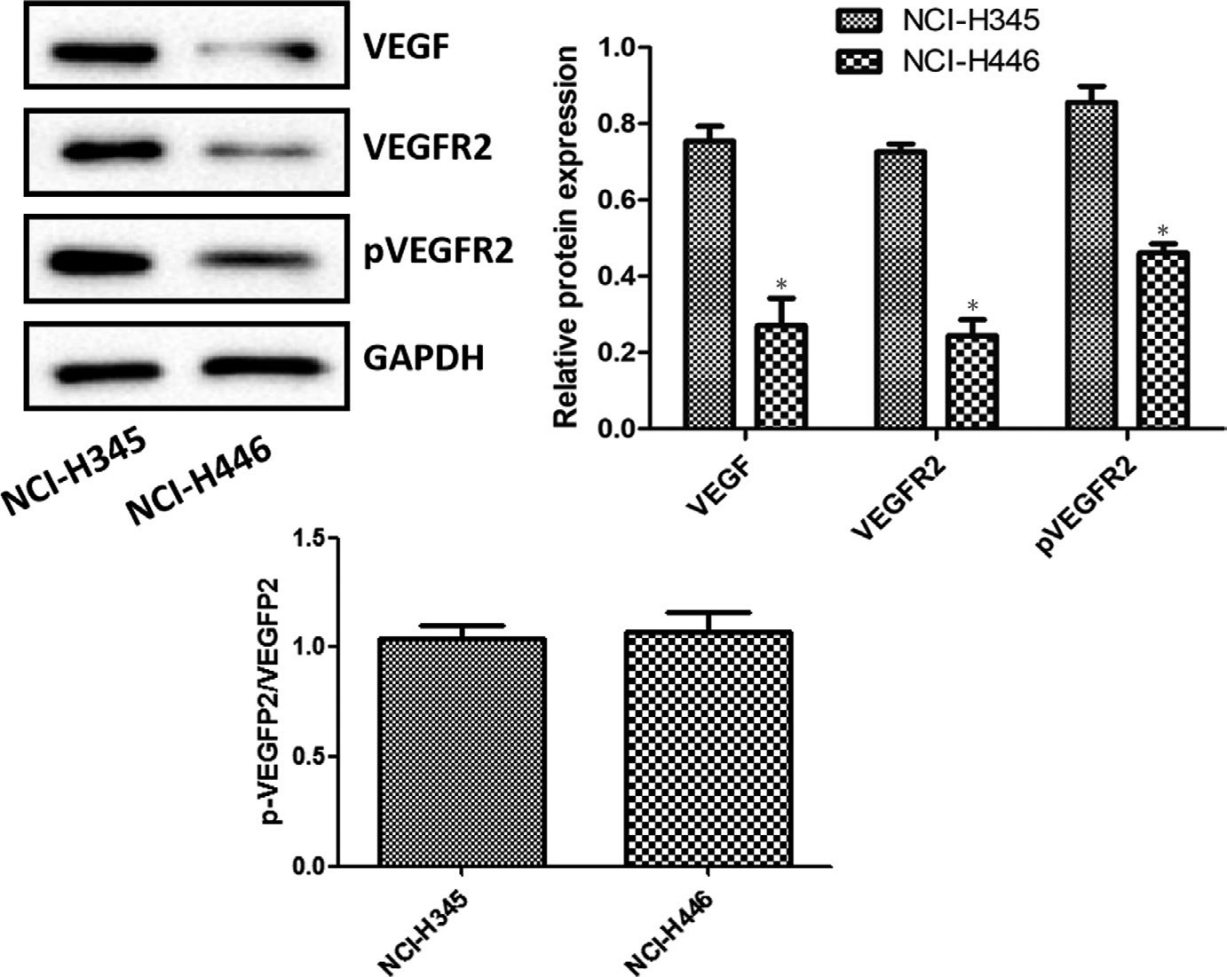
Levels of VEGF, VEGFR2 and pVEGFR2 in NCI‐H345 and NCI‐H446 cells which were examined by Western blotting. All blots were normalized to GAPDH levels. ^*^ *p* < 0.05 vs. NCI‐H345 cells. VEGF, vascular endothelial growth factor; VEGFR2, vascular endothelial growth factor receptor 3; p, phosphorylated

**FIGURE 2:**
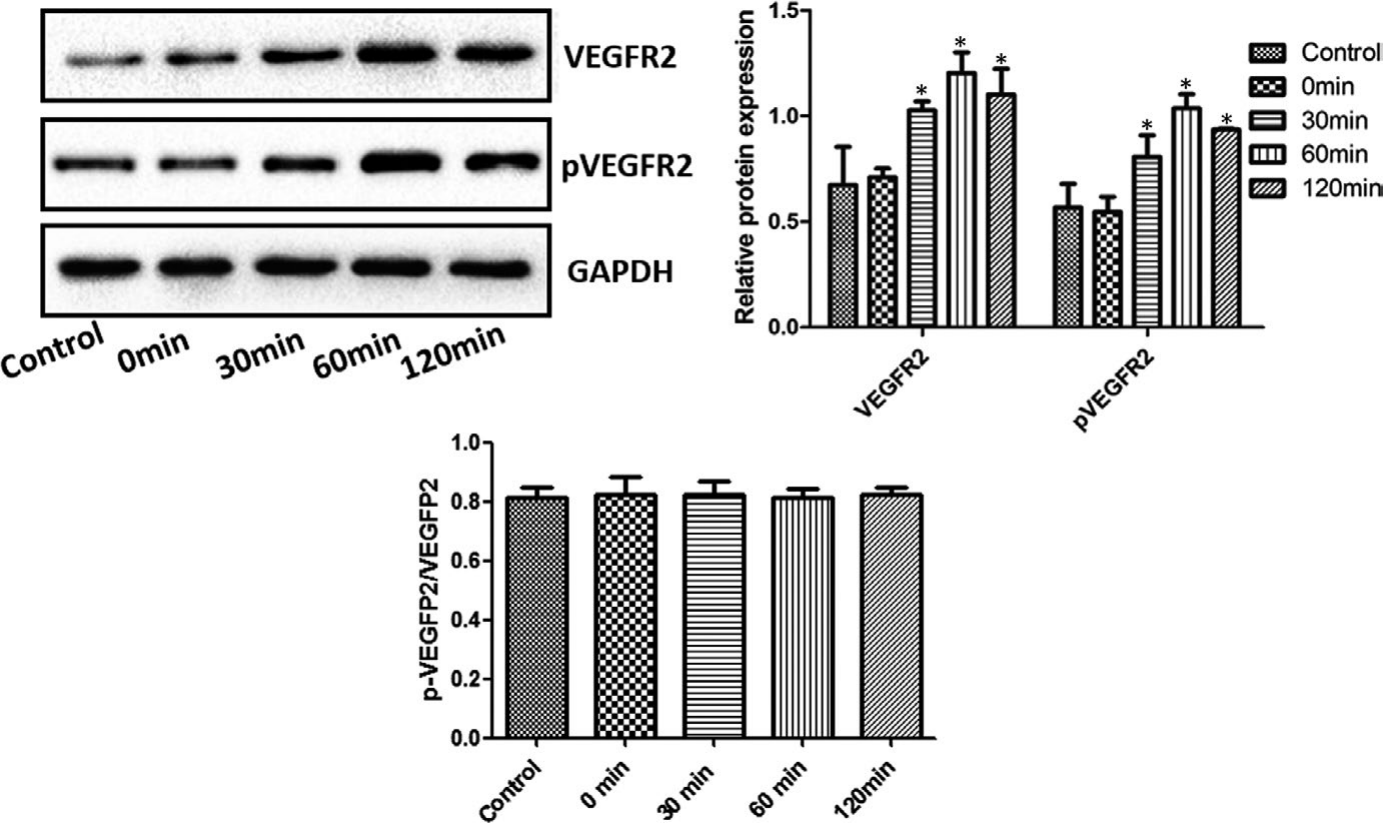
Levels of VEGFR2 and pVEGFR2 in NCI‐H345 cells following treatment with treatment with rhVEGF for 0, 30, 60 and 120 min analysed by Western blotting. All blots were normalized to GAPDH levels. ^*^ *p* < 0.05 vs. control. VEGFR2, vascular endothelial growth factor receptor 3; p, phosphorylated; rhVEGF, recombinant human VEGF

The levels of VEGF, VEGFR2 and pVEGFR2 in NCI‐H345 and NCI‐H446 cells were assessed by Western blot analysis (Figure 1). Although the ratio of pVEGFR2 versus VEGFR2 was similar between the two cells, compared with NCI‐H345 cells, the expression of VEGF, VEGFR2 and pVEGFR2 in NCI‐H446 cells was significantly reduced (*p* < 0.05). Therefore, NCI‐H345 cells were selected for experimentation. The levels of VEGFR2 and pVEGFR2 in NCI‐H345 cells following treatment with rhVEGF for 0, 30, 60 and 120 min were investigated by Western blot analysis (Figure 2). The ratio of pVEGFR2 versus VEGFR2 was similar among various treatments. However, the expression levels of VEGFR2 and pVEGFR2 in rhVEGF‐treated NCI‐H345 cells were significantly upregulated compared with the controls (*p* < 0.05). Considering that the expression levels of VEGFR2 and pVEGFR2 were the highest at 60 min, this time point was selected for experimentation.

### 3.2 Cell proliferation in vitro

**FIGURE 3:**
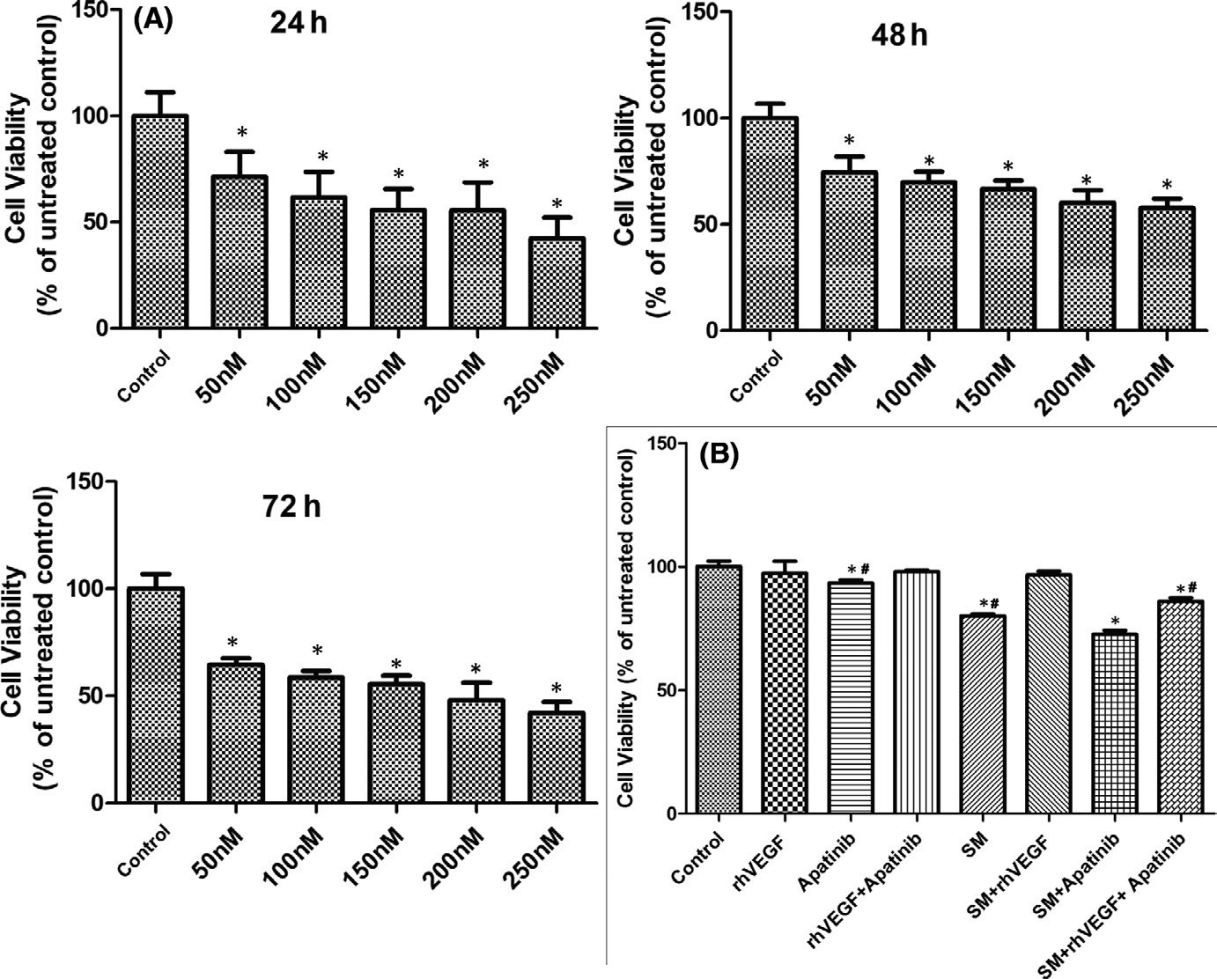
The proliferation rate of NCI‐H345 cells (A) following treatments with increasing concentrations of apatinib (50, 100, 150, 200 and 250 nM) for different durations (24, 48 and 72 h) and (B) after various treatments of rhVEGF, apatinib and serum‐free medium for 60 min, as evaluated by Cell Counting Kit‐8 assay. ^*^ *p* < 0.05 vs. control; ^#^ *p* < 0.05 vs. SM+apatinib. SM, serum‐free medium

The proliferation rate of NCI‐H345 cells treated with different concentrations of apatinib (50, 100, 150, 200 and 250 nM) for different times (24, 48 and 72 h) is presented in Figure 3A. Apatinib reduced cell proliferation at all concentrations and time points. Moreover, the cell viability decreased with increasing concentration of apatinib. Therefore, the highest concentration of apatinib (250 nM) was selected for subsequent experimentation. However, considering that a long exposure time may reduce cell proliferation and affect the results, an exposure time of 60 min was selected.

The proliferation rate of NCI‐H345 cells in the eight groups was evaluated by the CCK‐8 assay (Figure 3B). The cells in the control, rhVEGF, rhVEGF+apatinib and serum‐free medium+rhVEGF groups exhibited similar proliferation rates (*p *> 0.05). In comparison with the control group, the proliferation rate of cells in the apatinib, serum‐free medium, serum‐free medium+apatinib and serum‐free medium+rhVEGF+apatinib groups significantly decreased, particularly in the serum‐free medium+apatinib group (*p* < 0.05).

### 3.3 Tumour growth in vivo

**FIGURE 4:**
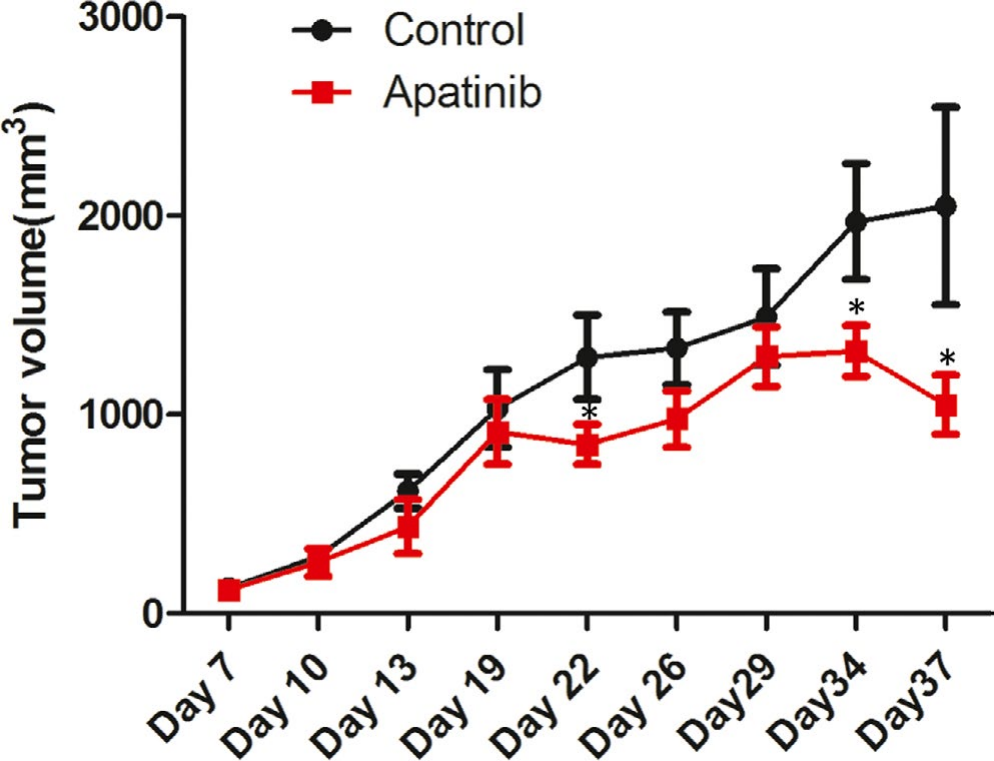
Tumour size‐time curve exhibiting tumour growth in a tumour xenograft model in apatinib‐treated and control mice. ^*^ *p* < 0.05 vs. control

Tumour growth in mice following apatinib treatment was investigated using a tumour xenograft model. Multiple tumours were not presented in all mice. The tumour size‐time curve is presented in Figure 4. In comparison with the control group, tumour sizes in the apatinib‐treated mice were significantly reduced 34 and 37 days after cell injection (*p* < 0.05). The tumour volume decreased on day 37.

### 3.4 Expression of VEGF, pVEGFR2, PI3K, p‐PI3K, AKT, p‐AKT, ERK1/2, p‐ERK1/2 and Ki‐67

**FIGURE 5:**
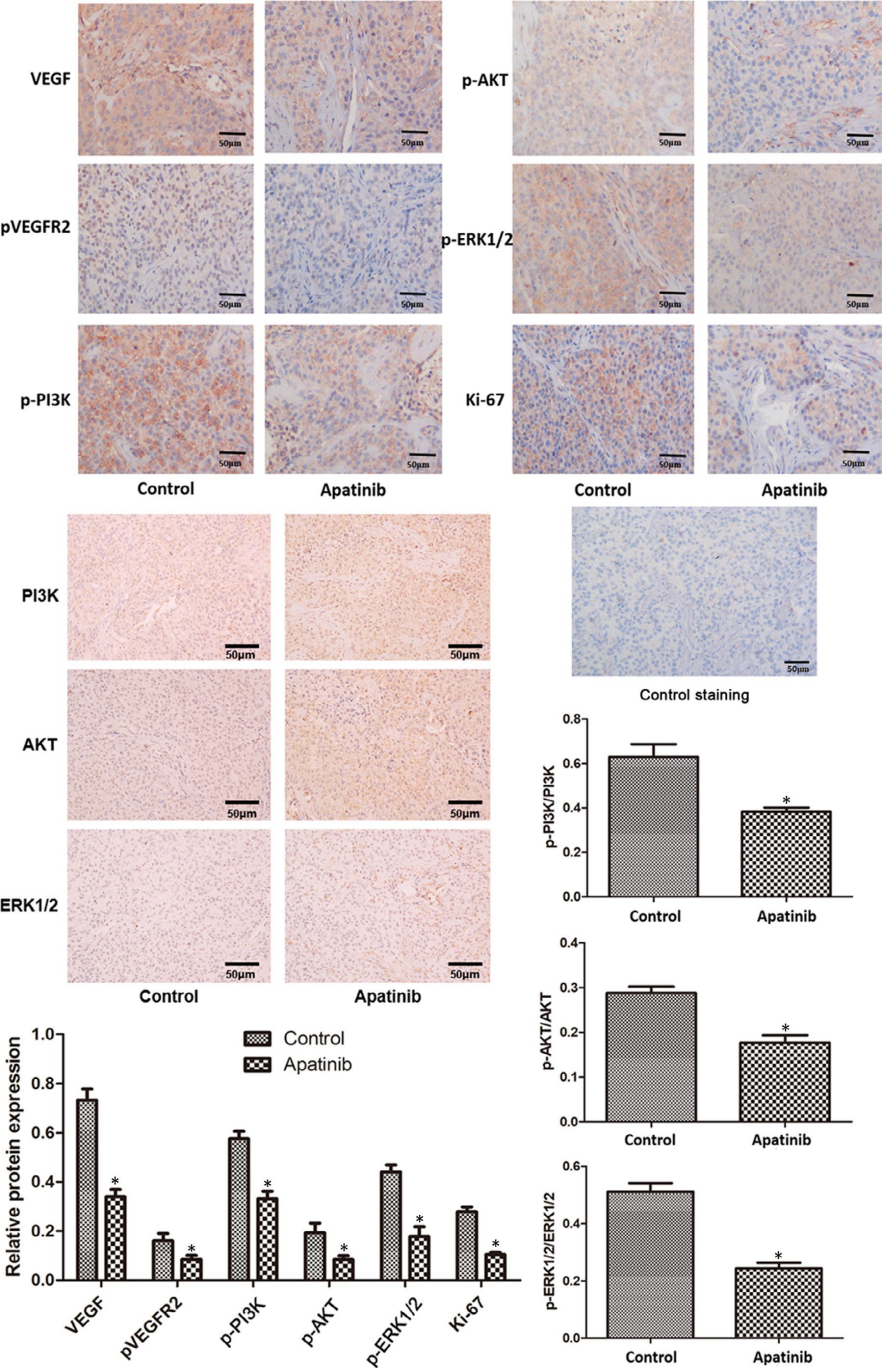
Expression of VEGF, pVEGFR2, PI3K, p‐PI3K, AKT, p‐AKT, ERK1/2, p‐ERK1/2 and Ki‐67 in tumour tissues was determined by immunohistochemical analysis. Negative control staining was performed using irrelevant isotype antibody. The control images were obtained from the cells/tissues of control animals. The control staining image was the negative control, which was used to exclude the non‐specific reaction of the antibody. ^*^ *p* < 0.05 vs. control. VEGF, vascular endothelial growth factor, p, phosphorylated; VEGFR2, vascular endothelial growth factor receptor 2; PI3K, phosphoinositide 3‐kinase; AKT, protein kinase B; Ki‐67, marker of proliferation Ki‐67

The expression level of VEGF, pVEGFR2, PI3K, p‐PI3K, AKT, p‐AKT, ERK1/2, p‐ERK1/2 and Ki‐67 in tumour tissues harvested from the xenograft model was determined by immunohistochemical analysis (Figure 5). In comparison with the mice in the control group, the expression levels of VEGF, pVEGFR2, p‐PI3K, p‐AKT, p‐ERK1/2 and Ki‐67 in tumours obtained from apatinib‐treated mice were significantly downregulated (*p* < 0.05). Negative control staining by using an irrelevant isotype antibody was performed to ensure that the staining for the markers of interest was specific.

### 3.5 CD31 expression

**FIGURE 6:**
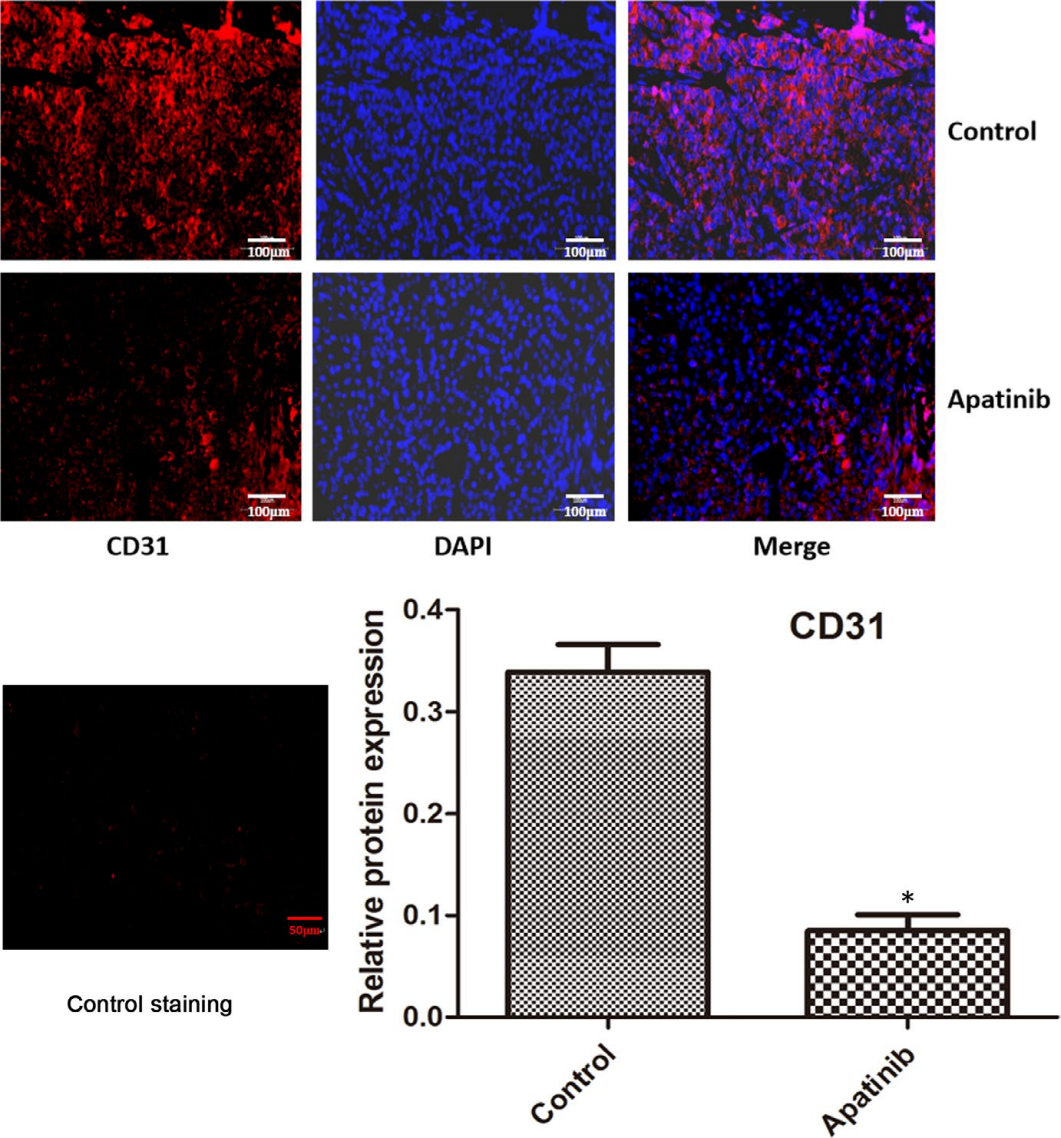
CD31 expression in tumour tissues was analysed by immunofluorescence staining. CD31 protein is presented in red and DAPI in blue. Negative control staining was performed using irrelevant isotype antibody. The control images were obtained from the cells/tissues of control animals. The control staining image was the negative control, which was used to exclude the non‐specific reaction of the antibody. ^*^ *p* < 0.05 vs. control. CD31, CD31 antigen

The expression level of CD31 in tumour tissues harvested from the xenograft model was determined by immunofluorescence analysis (Figure 6). In comparison with the mice in the control group, the expression level of CD31 in tumours obtained from apatinib‐treated mice was significantly downregulated (*p* < 0.05). Negative control staining by using an irrelevant isotype antibody was performed to ensure that the staining for CD31 was specific.

### 3.6 Haematoxylin and eosin staining

**FIGURE 7:**
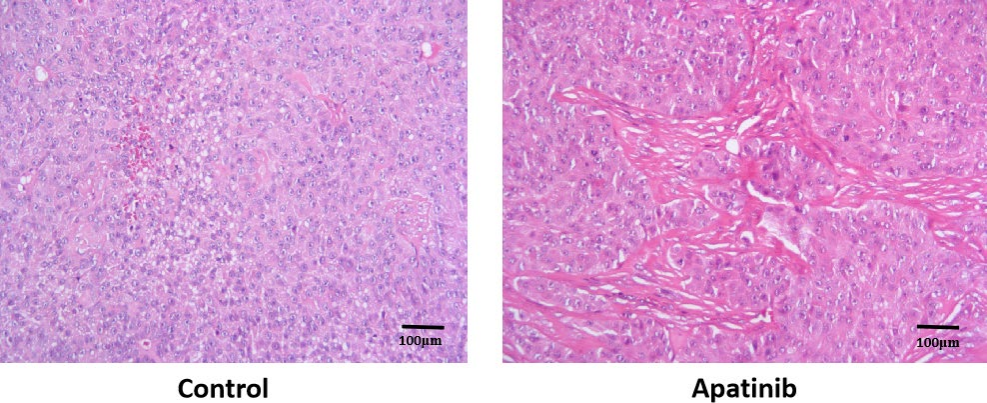
Representative haematoxylin and eosin staining of tumour tissues obtained from apatinib‐treated mice

Representative haematoxylin and eosin staining images of the tumour tissues obtained from apatinib‐treated mice are presented in Figure 7. The control group exhibited a uniform distribution of tumour cells and absence of necrotic areas. However, following treatment with apatinib, large areas of necrosis in the tumour tissues were observed.

## 4 DISCUSSION

Apatinib mesylate is a small molecule VEGFR2 tyrosine kinase inhibitor developed in China that can be administered orally. Apatinib competitively binds to the adenosine triphosphate binding site in VEGFR2, thus blocking the downstream signalling pathways and inhibiting tumour angiogenesis.^18^, ^19^, ^20^, ^21^ VEGF has an angiogenesis‐promoting effect.^22^ Additionally, the VEGF autocrine signalling pathway in tumour cells plays an important role in tumour growth.^22^ Tumour cells in different types of cancer, including cholangiocarcinoma, hepatocellular carcinoma and NSCLC,^23^, ^24^, ^25^ co‐express VEGF and VEGFR2, and the interaction between these two proteins promotes the proliferation of tumour cells.^26^

The present study revealed that apatinib inhibited SCLC cell proliferation *in vitro*, and this effect was inhibited by exogenous rhVEGF. In addition, tumour sizes in apatinib‐treated mice apatinib were smaller than those of control mice. Therefore, apatinib inhibited the growth of SCLC tumours *in vitro* and *in vivo*. The tumour volume on day 37 decreased possibly because the effect of apatinib on the tumour became increasingly obvious with prolonged administration time. Additionally, the expression levels of VEGF and pVEGFR2 in tumour tissues were downregulated following apatinib treatment, suggesting that apatinib inhibited the expression of VEGF and pVEGFR2 in SCLC tumours.

The PI3K/AKT signalling pathway is involved in tumorigenesis and activates the expression of downstream genes or proteins to induce cell proliferation, regulate downstream apoptotic or anti‐apoptotic genes, mediate haematopoiesis and angiogenesis, and increase oxygen utilization by tumour cells by regulating glucose transport and glycolysis‐associated enzymes.^27^ ERK1/2 is a member of the mitogen‐activated protein kinase family.^28^ The activation of ERK1/2 signalling pathway is closely associated with the development and progression of various tumours, such as renal cancer, hepatocellular carcinoma, prostatic carcinoma and NSCLC.^29^ Apatinib does not affect the expression of total PI3K, AKT and ERK1/2.^17^, ^30^ The expression of p‐PI3K and p‐AKT in SCLC tumours was remarkably decreased following apatinib treatment, suggesting that apatinib may inhibit SCLC tumour growth by inhibiting the PI3K/AKT signalling pathway. The results of the present study revealed that the expression levels of p‐ERK1/2, Ki‐67 and CD31 in SCLC tumours were significantly reduced following apatinib treatment, suggesting that apatinib may also inhibit SCLC tumour growth by inhibiting the expression of p‐ERK1/2, Ki‐67 and CD31. ERK1/2 is activated in NSCLC and is associated with advanced tumours.^29^ A limitation of the present study is that Western blot experiments were not conducted to determine the levels of the phosphorylated and total proteins mentioned above. Ki‐67 is a nucleoprotein involved in ribosomal RNA transcription and is expressed at all stages of cell proliferation, except the G0 stage.^31^ Ki‐67 inactivation inhibits ribosomal RNA synthesis, and Ki‐67 is used as a biomarker of cell proliferation.^31^ The Ki‐67 index in pathological reports is closely associated with the differentiation, invasion, metastasis and prognosis of tumours, such as cervical cancer.^32^ Increased Ki‐67 expression in tumours is associated with increased degree of malignancy, increased tendency for invasion and metastasis, and poor prognosis.^33^ CD31 is mainly used to identify endothelial cells and evaluate tumour angiogenesis, which may indicate the rate of tumour growth.^34^ Malignant vascular endothelial cells generally retain antigens, thus allowing the use of CD31 expression to diagnose haemangioma and angiosarcoma.^34^

The present study mainly aimed to determine the anti‐tumour effect of apatinib on ES‐SCLC, but the research on related mechanisms is not systematic enough. Only the effects on some pathways or indicators related to tumour are revealed. We will further explore the effects of apatinib on various solid tumours and determine the effect of apatinib on the PI3K/AKT signalling pathway.

In conclusion, the present study revealed that apatinib inhibited SCLC tumour growth *in vitro* and *in vivo* by downregulating the expression of VEGF, pVEGFR2, p‐PI3K, p‐AKT, p‐ERK1/2, Ki‐67 and CD31. These results suggest that the potential application of apatinib for the treatment of ES‐SCLC requires further investigation and may aid in the identification of novel therapeutic targets and diagnostic markers.

## CONFLICT OF INTEREST

The authors confirm that there are no conflicts of interest.

## AUTHOR CONTRIBUTIONS

**Ning Zhong:** Conceptualization (equal); Data curation (equal); Formal analysis (equal); Investigation (equal); Methodology (equal); Validation (equal); Writing‐original draft (equal); Writing‐review & editing (equal). **Wei Zhuang:** Conceptualization (equal); Data curation (equal); Formal analysis (equal); Investigation (equal); Methodology (equal); Validation (equal); Writing‐original draft (equal); Writing‐review & editing (equal). **Qian Huang:** Data curation (supporting); Formal analysis (supporting); Investigation (supporting); Methodology (supporting). **Qiang Wang:** Data curation (supporting); Formal analysis (supporting); Investigation (supporting); Methodology (supporting). **Wenjian Jin:** Conceptualization (lead); Funding acquisition (lead); Project administration (lead); Resources (lead); Supervision (lead); Visualization (lead); Writing‐original draft (equal); Writing‐review & editing (equal).

## Data Availability

The analysed data sets generated during the study are available from the corresponding author on reasonable request.
